# Successful treatment of Fusarium solani anterior chamber involvement secondary to contact lens associated corneal ulcer with intracameral Amphotericin B

**DOI:** 10.3205/oc000092

**Published:** 2019-02-12

**Authors:** Patrick Peters, Isabel Oberacher-Velten, Horst Helbig, David Märker

**Affiliations:** 1 Department of Ophthalmology, University Hospital Regensburg, Germany

## Abstract

**Background:** *Fusarium* spp. are leading fungal pathogenes in contact lens associated keratitis and may evoke endophthalmitis. Since *Fusarium* spp. are highly resistant to antifungal drugs, globe integrity is threatened.

**Case:** A woman developed fungal anterior chamber involvement after contact lens associated corneal ulcer formation. She presented with a painful eye with hypopyon and a mass presumably of fungal origin growing on the iris and anterior lens capsule. A biopsy confirmed *Fusarium solani*. Only multiple lavages of the anterior segment with Amphotericin B achieved convalescence.

**Conclusion:** In the initial stage of contact lens associated keratitis, it is often hard to differentiate between etiology. However, keratitis which are treatment resistant to antibacterials are suspicious for fungal origin. For proper treatment, identification of pathogen is crucial. Due to poor tissue penetration of the lipophilic anti-fungal agents and slow fungal replication rate, multiple lavages of the anterior chamber are often required to handle fungal infections.

## Case description

**Figure 1:**
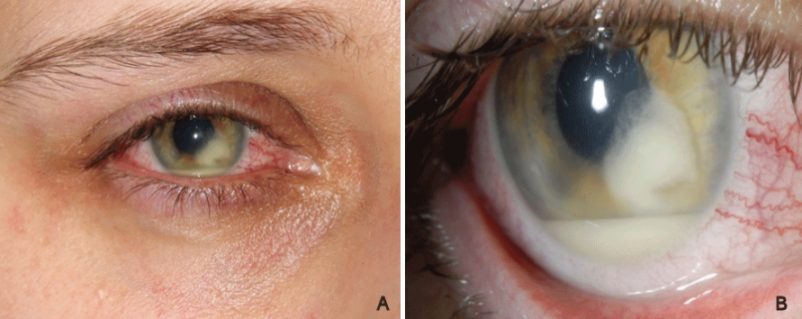
Slit-lamp photo of the right eye showing hypopyon and fungal mass in the anterior chamber (day 1)

**Figure 2:**
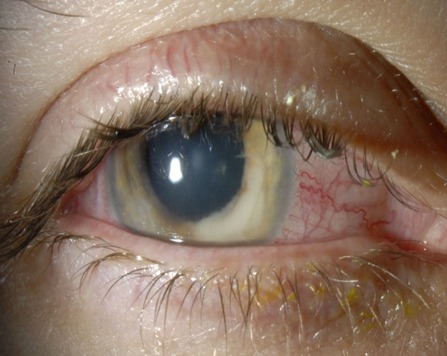
Examination following first anterior chamber lavage (day 3)

A 28-year-old female patient presented with a medical history significant for contact lens associated keratitis. She had been treated with several different topical antibiotics for almost three months due to contact lens associated corneal infiltration. Due to increasing anterior chamber involvement, local and systemic steroids had been added after one month. Since the patient’s symptoms failed to improve, she was referred to our clinic. Best corrected visual acuity was 8/20 on her right eye and 20/20 on her left. Intraocular pressure was normal. Slit lamp examination revealed on the right eye a peripheral corneal infiltration with a central ulcer, a deep anterior chamber with a mass on the iris and a hypopyon (Figure 1 (Fig. 1)). These features evoked suspicion of a fungal intraocular infection. Steroids were stopped and a biopsy of the mass was performed with bimanual irrigation and aspiration handpiece use (Figure 2 (Fig. 2)). Topical treatment with Natamycin 5% and Voriconazole 1.9% eye drops was started hourly, and initially Voriconazole 400 mg intravenously was also given twice a day. First specimen taken showed *Fusarium* spp. without further subdifferentiation. Intracameral lavage with Amphotericin B (7.4 µg) was performed by tap and inject in the operating room. A second specimen was taken with bimanual irrigation and aspiration handpiece use for further subdifferentiation and antifungal drug susceptibility testing after 3 injections of Amphotericin B.

**Figure 3:**
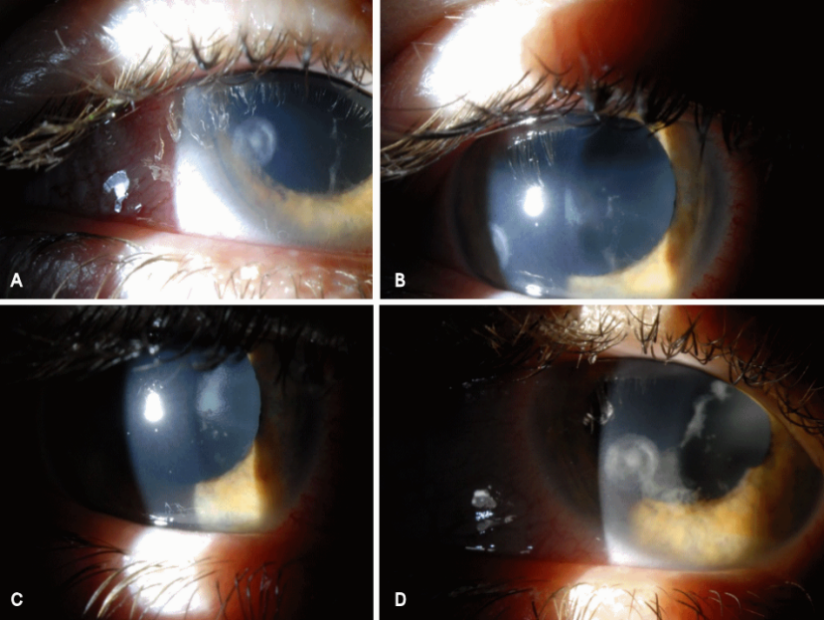
Progressive resolution of fungal mass during intracameral Amphotericin B treatment on day 7 (A/B), day 11 (C), and day 15 after its discontinuation (D)

Initially, anterior chamber lavage was performed once a day for four consecutive days (Figure 3A,B (Fig. 3)). Two days later, slit lamp examination revealed small whitish dots on the anterior lens capsule (Figure 3C (Fig. 3)). Assuming persistent activity of intraocular infection lavage of the anterior chamber with Amphotericin B was continued once a day for another five days.

*Fusarium solani* was isolated from the second specimen and sent to a reference laboratory to perform a Sensititre YeastOne microdilution antifungal susceptibility testing. The result took seven days. Amphotericin B had the highest efficacy (2 µg/ml), followed by Natamycin (4 µg/ml), whereas Azoles (Voriconazole, Posaconazole, Itraconazole) were ineffective. Consequently, topical and systemic therapy with Voriconazole was discontinued. At no time did the vitreous or retina show signs of infiltration. Topical treatment was gradually tapered to Natamycin 5 times daily.

**Figure 4:**
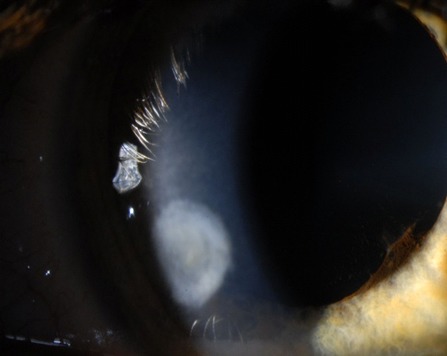
Result on last follow-up visit six months later

The patient was discharged after two weeks of aggressive treatment with easing of local signs (Figure 3D (Fig. 3)). After six months, her visual acuity has improved to 200/200 on the right eye and slit lamp examination revealed a cicatrizing peripheral corneal ulcer with closed epithelium (Figure 4 (Fig. 4)).

## Discussion

Leading transmission pathways in fungal infections include hematogenous dissemination, open globe injury, or injuries with organic matter, contamination during surgery, or insufficient hygiene precautions with contact lenses [1]. Fungal infections of the eye ball often present initially with contact lens associated keratitis [2]. In contrast to bacterial keratitis, fungi can penetrate Descemet’s membrane and infiltrate the anterior chamber where most of the fungal infections take place [3]. When defense mechanisms are impaired by steroids and immunosuppressive agents, such as in the presented case, the risk of invasive fungal infections is enhanced [4]. Features of intraocular fungal infections are endothelial plaque, hypopyon, fungal web or fungal ball in the anterior chamber [5]. Eradication of invasively growing fungi is a challenging task.

Commonly isolated pathogens are *Fusarium* spp. as well as *Candida* spp. and *Aspergillus* spp. Their natural habitat is in ambient air and water. *Fusarium* species are known to have a high drug resistance potential. They have the ability to secrete extracellular proteases which cause tissue degradation and enhance invasive growth [6].

Topical Natamycin is the first-line therapy in filamentous fungal keratitis. However, due to its high hydrophobic characteristics, Natamycin is unable to penetrate into the anterior chamber or vitreous. Therefore, its use is limited to corneal treatment [7]. Since clinicians have most experience with Amphotericin B, one of the oldest antifungal drugs, it is mostly used in combination with other agents until microbial test results are available [2]. In contrast to Natamycin, Amphotericin B can be applied by multiple routes including systemic, topical, subconjunctival, intravitreal, and intracameral administration. Furthermore, since aqueous clearance of intracamerally administered Amphotericin B is quite high, repeated injections are needed to maintain a constant, effective drug concentration [8]. Dosages ranging from 5 to 10 µg in 0.1 mL seem to be safe for intravitreal administration without adverse events.

Although a large body of experiences with Amphotericin B has been collected, the potential risk for endothelial and lens toxicity should be considered. Yilmaz et al. [9] reported a case of cataract development after intracameral Amphotericin injection. It is presumed that the anterior lens capsule was involved in the infection. After anterior chamber lavage, iris hyperemia, pain, and severe reactions of the anterior segment have been reported.

The main difficulty in fungal eye infections is to verify the responsible pathogen to prevent further loss of vision or even loss of the eye ball [10]. Fast determination of the responsible pathogen and consequent treatment after performing a Sensititre YeastOne microdilution antifungal susceptibility test appears to be the only way to prevent false treatment [11]. Most *Fusarium* spp. show resistance to several antifungal agents with the notable exception of Amphotericin B [6]. In our case, intracameral treatment with Amphotericin B was the right choice and systemic treatment with Voriconazole was stopped.

Compared to other reports, we present a well photodocumented case of a Fusarium endophthalmitis secondary to corneal ulceration treated with intracameral Amphotericin B. Our treatment regime demonstrates effectiveness in Azole resistant *Fusarium solani*.

In summary, we present a case of successfully treated fungal infection caused by contact lens associated keratitis with preservation of the globe and good visual acuity. Final visual function in contact lens associated fungal keratitis depends on the localization of the remaining corneal scar. It can be necessary to perform a perforating keratoplasty as secondary step to improve vision. In any case, an aggressive composite treatment is needed, after rapid identification of the responsible pathogen to ensure globe integrity.

## Notes

### Competing interests

The authors declare that they have no competing interests.
